# Bortezomib in Patients Who Fail BTKi in Waldenström Macroglobulinaemia

**DOI:** 10.3390/hematolrep18030036

**Published:** 2026-05-30

**Authors:** Jahanzaib Khwaja, Nicole Japzon, Simona Gatto, Jindriska Lindsay, Charalampia Kyriakou, Shirley D’Sa

**Affiliations:** 1 Department of Haematology, University College London Hospital, London NW1 2BU, UK; nicole.japzon@nhs.net (N.J.); charalampia.kyriakou1@nhs.net (C.K.);; 2 Department of Haematology, University Hospital of Wales, Cardiff CF14 4XW, UK; simona.gatto@wales.nhs.uk

**Keywords:** Waldenström macroglobulnaemia, BTKi, bortezomib

## Abstract

**Background:** Treatment options after Bruton’s tyrosine kinase inhibitors (BTKi) failure in Waldenstrom macroglobulinaemia are limited. **Methods:** We retrospectively analysed the use of bortezomib after BTKi failure in 17 patients who were heavily pre-treated and chemotherapy-exposed at our centre between 2018 and 2025. **Results:** Reasons for BTKi failure were disease progression (59%) and intolerance (41%). At bortezomib initiation, the median age was 73 years and two patients experienced grade 1–2 neuropathy. The best overall response rate (ORR) was 88%. At a median follow up of 39 months (interquartile range 35–78), median treatment-free survival and overall survival were 18 (95% confidence interval [CI] 13–22) and 22 (95% CI 17–45) months, respectively. **Conclusion:** Bortezomib may be efficacious in patients who experience BTKi failure.

## 1. Introduction

Waldenstrom macroglobulinaemia (WM) is an indolent, incurable lymphoma characterised by an IgM monoclonal protein and lymphoplasmacytic bone marrow infiltrate with a median survival of a decade [1]. Bruton’s tyrosine kinase inhibitors (BTKi) have become a cornerstone of therapy as these are effective and tolerable and may be used in frailer patients or those unsuitable for chemoimmunotherapy. Oral continuous covalent BTKi are FDA-approved, although reimbursement is country-specific. In the United Kingdom (UK) and Europe, fixed-duration chemoimmunotherapy (bendamustine–rituximab [BR], dexamethasone–rituximab–cyclophosphamide [DRC]) remains largely employed in the first-line setting and BTKi at relapse. However, treatment options after BTKi failure are limited; there is no consensus regarding sequencing at this juncture.

Bortezomib, a first-generation proteasome inhibitor, targets regulatory proteins contributing to tumour genesis in plasma cell dyscrasias, and has shown efficacy in WM [2,3] and may be considered an option for relapse or refractory WM [4]. Scant data, however, are available regarding bortezomib use after BTKi failure. We previously reported on the real-world use of bortezomib in the UK [5]. Here, we updated this cohort and analysed the use of bortezomib combination in patients after chemotherapy exposure and BTKi ‘failure’ in an expanded, updated cohort.

## 2. Methods

Consecutive patients treated with bortezomib combination therapy for relapsed WM after prior BTKi exposure from June 2018 to June 2025 at our referral centre, University College London Hospitals, were retrospectively reviewed. Electronic records for all patients were interrogated for baseline characteristics and outcomes, in accordance with IWWM-11 consensus criteria [6]. Treatment decisions, including dosing and frequency, were at the discretion of the treating physician as a part of routine care. *MYD88* mutation testing was performed by real-time PCR (Plentiplex assay, PentaBase, Odensef C, Denmark) and *CXCR4* mutation by next-generation sequencing on unselected cells (Archer Myeloid VariantPlex assay, Integrated DNA Technologies, Coralville, IA, USA) with a sensitivity of 0.25 and 1%, respectively. Toxicity was graded using the Common Terminology Criteria for Adverse Events (CTCAE) version 6.0 (The National Cancer Institute, Bethesda, MD, USA). Overall survival (OS) and treatment-free survival (TFS) were defined as time from bortezomib initiation to death from any cause and next-line therapy/death or last follow up, respectively. Survival estimates were generated using the Kaplan–Meier method. All statistical analyses were conducted using STATA v18 (StataCorp LLC, College Station, TX, USA).

## 3. Results

Of 45 patients receiving at least one dose of bortezomib for relapse WM, 17 (14 male; 3 female) had prior BTKi exposure, and outcomes are reported here. The median age at WM diagnosis was 64 years (range 41–86); in 72% (8/11) *MYD88 L265P* and 17% (1/6), *CXCR4 S338X* mutations were detected. First-line therapy included BR or DRC (*n* = 5 each) in most and the remaining underwent R-CHOP-like (*n* = 4), chlorambucil + rituximab (2; 13%) and fludarabine-based (*n* = 1) therapies.

Baseline characteristics at the time of bortezomib administration are outlined (Table 1). A median of four lines of therapy (range 2–6) were delivered prior to bortezomib. None had received a proteasome inhibitor previously. All patients were BTKi-exposed: mostly ibrutinib (14; 82%), zanubrutinib and acalabrutinib (2; 12% each, including one after ibrutinib) and two received pirtobrutinib after ibrutinib failure. The median duration of BTKi therapy was 11 months (interquartile range [IQR] 5–18). Reasons for BTKi failure were disease progression in 10 patients (59%) and drug intolerance in 7 (41%) (bleeding: cerebral *n* = 1; epistaxis *n* = 2; arthralgia/periarticular lymphoplasmacytic lymphoma infiltration or bleeding *n* = 3; cytopenias *n* = 1).

Time to next line of therapy from BTKi discontinuation was 3 months (95% CI 1–6). The median time from BTKi discontinuation to bortezomib was 7 months (IQR 3–20). At bortezomib initiation, the median age was 73 (range 46–89) years, haemoglobin 97 g/L (range 55–148), platelets 193 × 10^9^/L (range 7–518), neutrophils 3.6 × 10^9^/L (range 0.32–13.8), immunoglobulin G concentration 3.22 g/L (range <0.3–28.8), monoclonal IgM protein 28 g/L (range 4–75) and bone marrow trephine lymphoplasmcytoid infiltrate 45% (range 5–70). A total of 3/16 had platelet <50 × 10^9^/L and neutrophils <1.5 × 10^9^/L.

The following bortezomib-containing regimens were delivered: bortezomib–dexamethasone (8; 47%), bortezomib–dexamethasone–rituximab (5; 29%), bortezomib–dexamethasone–cyclophosphamide (3; 18%) and bortezomib–dexamethasone–daratumumab (1; 6%) at a subcutaneous dose of 1.3 mg/m^2^ in 15 (88%) and 1.6 mg/m^2^ in 2 (12%). Four patients (24%) experienced grade 3–4 neutropenia and two patients (12%) each experienced grade 3–4 thrombocytopenia (3/4 had ≥3 prior treatment lines) and grade 1–2 of new or worsening neuropathy. Neuropathy reduced to grade 1 after treatment discontinuation. No treatment-related deaths, cardiac or renal toxicities were documented.

**Figure 1:**
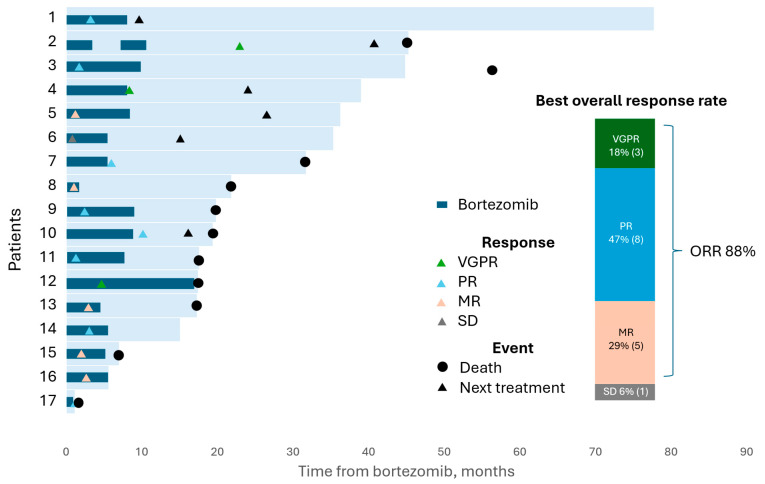
Responses of patients after bortezomib administration. VGPR, very good partial response; PR, partial response; MR, minor response; SD, stable disease; ORR, overall response rate (created using Microsoft Excel 365, Microsoft, Redmond, WA, USA).

Figure 1 displays treatment responses. Patient 2 experienced treatment cessation during the COVID pandemic and patient 12 proceeded with continuous maintenance therapy pending clinical trial entry, whilst all other patients received fixed-duration treatment. Patient 8 discontinued treatment after one month due to development of bladder cancer. Bortezomib was not thought to be contributary.

Best overall response rate (ORR) was 88% and major response rate was 59%. Very good partial response was achieved in 3 (18%), partial response in 7 (41%), minor response in 5 (29%) and stable disease in 2 (12%), with a median time of 3 months (95% CI 1–4) required to achieve best response. At a median follow-up of 39 months (IQR 35–78) from bortezomib initiation, median TFS and OS were 18 (95% CI 13–22) and 22 (95% CI 17–45) months, respectively. Estimated 2-year TFS and OS were 27% (95% CI 8–50) and 47% (95% CI 22–69%), respectively. At data cut-off, 11 heavily pre-treated patients died due to disease progression (*n* = 7; 5 had ≥4 prior treatment lines), infection (*n* = 2; both ≥5 prior treatment lines), unrelated malignancy or other reasons (*n* = 1, each). Five underwent subsequent therapies, including further BTKi in two patients (nemtabrutinib; zanubrutinib), with ongoing responses.

**Figure 2:**
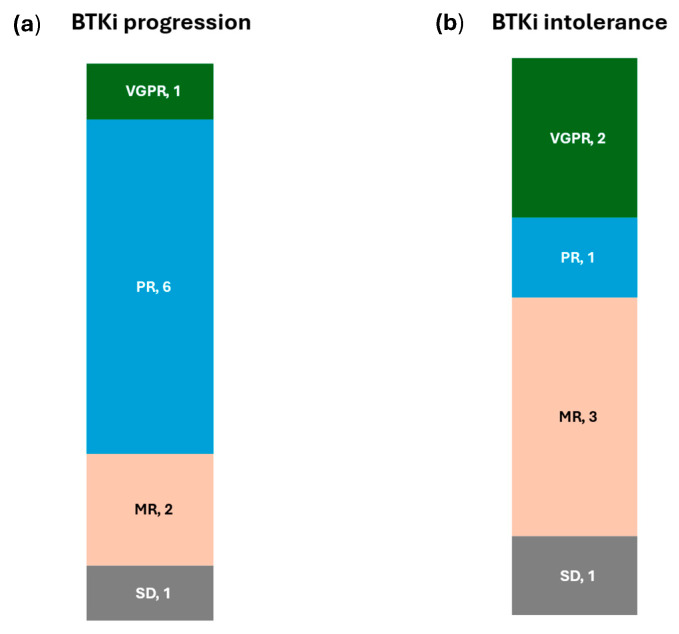
Responses of patients stratified by prior therapy: (**a**) progression on BTKi; (**b**) intolerance of BTKi. VGPR, very good partial response; PR, partial response; MR, minor response; SD, stable disease; ORR, overall response rate. (created using Microsoft Excel 365, Microsoft, Redmond, WA, USA).

Responses of both those who progressed on prior BTKi (*n* = 10) and those who were intolerant to BTKi (*n* = 7) are shown in Figure 2. Median OS was 20 months (95% CI 1–45) and 45 months (95% CI 7-NR) for BTKi progressors and BTKi-intolerant patients, respectively.

## 4. Discussion

There is no consensus on the optimal treatment for those with BTKi failure [7]. We present the largest series of bortezomib efficacy data in the setting of prior chemotherapy and covalent BTKi exposure due to disease progression or intolerance, such as life-threatening bleeding or severe or unmanageable side effects. Outcomes in heavily pre-treated patients are expectedly limited, as response durability declines with successive lines of therapy.

Whilst there are encouraging strategies to overcome such failure, such as non-covalent BTKi [8] and BTK degraders [9], these agents are limited or unavailable in many countries. The phase III ASPEN study reported lower adverse event-related discontinuation with zanubrutinib compared with ibrutinib [10]. Non-covalent BTKi, pirtobrutinib and nemtabrutinib may be used in those with acquired resistance to covalent BTKi as they bind BTK and C481S mutant BTK [8], with a phase I/II BRUIN study reporting 63/80 patients treated with pirtobrutinib with prior covalent BTKi exposure [11]. The management of patients after BTKi is an unmet need and the experience of 78 patients from 22 Italian centres after BTKi failure showed similar rates of progression and tolerance in the salvage setting. Bortezomib resulted in poorer outcomes, with 38% overall response and median progression-free survival of less than 6 months, albeit in an older population [12]. The median OS of the entire Italian relapse cohort was 21 months with a median of three prior lines of therapy.

Treatment approaches target the different cellular components in WM: the CD20 positive B cell-like component and CD20 negative plasma cell-like component responsible for IgM secretion. Recent studies identify molecular subtypes with distinct transcriptional and epigenetic regulation [13]. It has been reported that the B cell-like type is enriched with CXCR4 mutations and the plasma cell-like type is enriched with del6q [14,15]. Patients with the B cell-like type had higher circulating IgM concentration, splenic enlargement and less lymphadenopathy compared with the higher platelet count and lymphadenopathy in the plasma cell-like type. The molecular subtyping should be investigated in prospective studies. There is a known synergistic effect of bortezomib with dexamethasone [16]. Bortezomib selectively targets the plasma cell component, leading to more rapid IgM responses than traditional CD20-targeting strategies, DRC and BR, although recent data demonstrates that bone marrow B cell depletion independently predicts progression-free survival [17]. A flow cytometry assay was used in this study to detect WM-specific B cells with phenotype CD22(+wk)/CD25 +/CD200(+wk)/IgM+/CD305-/CD185(+wk). A WM-specific plasma cell phenotype has not been defined and cannot be reliably distinguished from normal plasma cells.

Evidence in the first-line setting showed that the addition of bortezomib to standard DRC induced more rapid and deep responses (ECWM-1 study), suggesting its potential use where clinical urgency is required, such as in those with hyperviscosity or bulky disease [18]. Symptomatic hyperviscosity may manifest with headaches, mucosal bleeding or neurological deficit and requires rapid intervention to avert major morbidity. Plasma exchange is utilised as a temporising measure, pending definitive clonal responses to chemoimmunotherapy. In addition, deep rapid responds are required for systemic AL amyloidosis and light chain deposition disease, and so are recommended as an option here [19].

Traditionally there have been concerns regarding neurotoxicity with intravenous bortezomib and administration twice a week; however, we report no grade 3–4 neuropathy in our selected cases, in which treatment was predominantly given subcutaneously at doses of 1.3 mg/m^2^. Toxicity was manageable and no treatment cessation occurred, nor was there clear signal of increased risk of secondary malignancy. Contrary to evidence published when bortezomib was administered at a higher dose (1.6 mg/m^2^) via the intravenous route, our data show good tolerability, especially in regard to neuropathy, which was minimal in this cohort. Other attractive features of bortezomib treatment include low cost, a treatment of fixed duration, low risk of myelosuppression and the possibility of home delivery and self-administration.

The optimal sequencing of bortezomib-based therapy in the relapse-refractory landscape remains uncertain. In our heavily pretreated cohort, including those traditionally excluded from clinical trials due to cytopenias (3/16: platelets < 50 × 10^9^/L and neutrophils <1.5 × 10^9^/L), the median duration of prior BTKi therapy was only 11 months, which is shorter than that typically observed in those with WM, suggesting our population was enriched aggressive disease or significant toxicity. Despite this, patients achieved an ORR of 88% and a reasonable treatment-free interval (median 18 months); treatment did not preclude future therapy, with two patients receiving further BTKi after bortezomib discontinuation. Fixed-duration treatment has been a focus of clinical trials due to its potentially reducing financial and long-term toxicities. The phase II trial of time-limited oral trials to reduce proteasome inhibitor ixazomib with ibrutinib included 12 relapsed patients with a recently reported ORR of 75% [20]. Fifty-nine patients receiving ixazomib–rituximab–dexamethasone with a median of two prior lines had a median duration of response of 36 months [21]. Other fixed-duration trials include novel drug combinations including Bcl-2 inhibition, such as pirtobrutinib–venetoclax (NCT05734495, recruiting), radiotherapeutic approaches such as Iopofosine I 131 (NCT02952508, completed recruitment) and CAR-T, although mature data is lacking. Thus far, bortezomib is an important option in the armamentarium of therapy post BTKi failure.

The limitations of this study include its retrospective nature and small sample size. Heterogenous bortezomib-based regimens were used, reflecting potential combination therapy rather than single-agent activity. There was no comprehensive evaluation of molecular studies performed at relapse with high sensitivity, which precluded assessment of clinical response by genotype, nor data on BTKi mutations, as this was not routinely performed.

## Figures and Tables

**Table 1:** Baseline characteristics at bortezomib administration.

	*n* = 17
Age at bortezomib administration (years), median (range)	73 (46–89)
Gender, *n* (%)	
Male	14 (82)
Female	3 (18)
Prior lines of therapy, median *n* (range/%)	4 (2–6)
BTKi	17 (100)
Ibrutinib	14 (82)
Acalabrutinib	2 (12)
Pirtobrutinib *	2 (12)
Zanubrutinib **	2 (12)
Bendamustine–rituximab	12 (71)
RCHOP/RCVP	6 (35)
Dexamethasone–rituximab–cyclophosphamide	5 (29)
Pembrolizumab	5 (29)
ESHAP/GDP	5 (29)
Fludarabine–cyclophosphamide–rituximab	3 (18)
Chlorambucil	2 (12)
Cladribine	1 (6)
Venetoclax	1 (6)
Iopofosine I 131	1 (6)
Bortezomib-containing regimen, *n* (%)	
Bortezomib–dexamethasone	8 (47)
Bortezomib–dexamethasone–rituximab	5 (29)
Bortezomib–dexamethasone–cyclophosphamide	3 (18)
Bortezomib–dexamethasone–daratumumab	1 (6)
Laboratory parameters, median (range)	
Haemoglobin, g/L	97 (55–148)
Platelets, ×10^9^/L	193 (7–518)
Neutrophils, ×10^9^/L	3.6 (0.32–13.8)
IgM monoclonal protein, g/L	28 (4–75)
Bone marrow lymphoplasmacytic infiltrate (%), median (range)	45 (5–70)

RCHOP, rituximab–cyclophosphamide–doxorubicine–vincristine–prednisolone; ESHAP, etoposide–methylpredisolone–high-dose cytarabine–cisplatin; GDP, gemcitabine–dexamethasone–cisplatin. * *n* = 1 pirtobrutinib after ibrutinib; ** *n* = 1 Zanubrutinib after ibrutinib.

## Data Availability

The authors confirm that the data supporting the findings of this study are available within the article. Additional data are available on request from the corresponding author.

## Abbreviations

The following abbreviations are used in this manuscript:

WM	Waldenstrom macroglobulinaemia
BTKi	Bruton’s tyrosine kinase inhibitor
ORR	Overall response rate
